# p40^*phox*^-Deficient Mice Exhibit Impaired Bacterial Clearance and Enhanced Pro-inflammatory Responses during *Salmonella enterica* serovar Typhimurium Infection

**DOI:** 10.3389/fimmu.2017.01270

**Published:** 2017-10-09

**Authors:** Yali Li, Meili Lv, Chienwen Su, Shaorong Long, Wei Zhang, Kara L. Conway, Weifen Li, Ramnik J. Xavier, Hai Ning Shi

**Affiliations:** ^1^Mucosal Immunology and Biology Research Center, Massachusetts General Hospital, Harvard Medical School, Boston, MA, United States; ^2^Zhejiang University College of Animal Sciences, Hangzhou, China; ^3^Animal Nutrition and Human Health Laboratory, School of Life Sciences, Hunan Normal University, Changsha, China; ^4^Sichuan University, Chengdu, China; ^5^Qinghai University Medical College, Xining, China; ^6^Gastrointestinal Unit, Center for the Study of Inflammatory Bowel Disease, Massachusetts General Hospital, Harvard Medical School, Boston, MA, United States

**Keywords:** NADPH oxidase, bacterial killing, mucosa, inflammation, *Salmonella* infection

## Abstract

*Salmonella enterica* serovar Typhimurium (*S*. Typhimurium) is a major cause of acute gastroenteritis in humans. During infection, reactive oxygen species (ROS), generated from NADPH oxidase (a multisubunit enzyme complex), are required for pathogen killing upon phagocytosis and for regulating pro-inflammatory signaling in phagocytic cells. Mutations in subunits forming the NADPH complex may lead to enhanced susceptibility to infection and inflammatory disease. Compared to other NADPH oxidase subunits, the function of p40*^phox^* is relatively understudied, particularly in the context of intestinal bacterial infection. In this study, we utilized genetically engineered mice to determine the role of p40*^phox^* in the response to *S*. Typhimurium infection. We show that mice lacking p40*^phox^* are more susceptible to oral infection with *S*. Typhimurium, as demonstrated by significantly enhanced bacterial dissemination to spleen and liver, and development of exacerbated bacterial colitis. Moreover, we demonstrate that the increased infection and disease severity are correlated with markedly increased F4/80^+^ macrophage and Ly6G^+^ neutrophil infiltration in the infected tissues, coincident with significantly elevated pro-inflammatory cytokines (IL-1β and TNF-α) and chemoattractant molecules in the infected tissues. Functional analysis of macrophages and neutrophils further shows that p40*^phox^* deficiency impairs bacteria- or PMA-induced intracellular ROS production as well as intracellular killing of *Salmonella*. These observations indicate that the p40*^phox^* subunit of NADPH oxidase plays an essential role in suppressing intracellular multiplication of *Salmonella* in macrophages and in the regulation of both systemic and mucosal inflammatory responses to bacterial infection.

## Introduction

*Salmonella enterica* serovar Typhimurium (*S*. Typhimurium) is a Gram-negative food-borne pathogen that is frequently associated with disease in various host species, including humans, livestock, domestic fowl, and rodents (1). In human, *S*. Tyhimurium is a major cause of acute gastroenteritis. There are approximately 40,000 cases of acute gastroenteritis annually in the United States (1). Although mice usually do not develop signs of the intestinal inflammation observed in humans following oral infection by *S*. Typhimurium, the bacteria penetrate the intestinal epithelium and move into the mesenteric lymph nodes (MLN), then spread to the circulatory system, causing systemic disease (2, 3). The *Salmonella*-induced intestinal inflammation model has also been established, which involves pretreatment of mice with streptomycin and then oral inoculation with a strain of *S*. Typhimurium that is naturally resistant to the antibiotic (4, 5). This bacterial colitis model provides an experimental system that is well suited to the analysis of host mucosal immune and inflammatory responses. In this model, replication of *S*. Typhimurium in lamina propria monocytic phagocytes/macrophages plays an important role in the development of colitis (6). However, it is less clear whether/how host mucosal defenses are affected by NADPH oxidase. In the infected intestinal mucosa, a mixture of different phagocytes has been observed, including polymorphonuclear neutrophils (PMN), dendritic cells (7), and macrophages, which play an essential role in host defense against invading bacterial pathogens. To effectively control the infection, molecules that can enhance the microbicidal capacity of phagocytes and the recruitment of various cell populations, such as neutrophils and monocytes, are required (2, 8, 9).

Phagocyte NADPH oxidase, a membrane-bound enzyme complex that is composed of six subunits, the catalytic subunit gp91*^phox^* and p22*^phox^*, and the cytosolic regulatory components (p47*^phox^*, p67*^phox^*, p40*^phox^*, and Rac), plays an important role in microbial killing through the generation of reactive oxygen species (ROS) (10–12). ROS generation is one of the central mechanisms used by phagocytes to defend against bacterial infection (13–15). The importance of ROS production in host defense is demonstrated by the enhanced susceptibility to infection of individuals who have inherited deficiencies of NADPH oxidase and develop chronic granulomatous disease (CGD) (16). gp91*^phox^* is the catalytic core of the NADPH oxidase and can produce ROS in the absence of other cytosolic subunits. Mutations in gp91*^phox^* account for the majority of CGD patients in the world (65% of all cases are the X-linked gp91*^phox^*-deficient form) (17). Evidence also shows that there is an excessive inflammation in CGD even in the absence of infectious agents (7). Furthermore, mice deficient in NADPH oxidase (*Cybb^−/−^*, encoding gp91*^phox^*) failed to control infection with a normally avirulent bacterial infection (6). Mutations in the *NCF4* gene encoding p40*^phox^*, a less well understood subunit of the NADPH oxidase complex, were first described in CGD with abnormal neutrophil function in 2009, which disproved the view that p40*^phox^* was not essential for NADPH oxidase activity (18). Evidence is also available to suggest a role for p40*^phox^*, in host resistance against *Staphylococcus aureus* infection and in the regulation of neutrophil recruitment and function (19, 20). In genetic studies the *NCF4* gene (p40*^phox^*) locus has been found to be associated with ileal and perianal Crohn’s disease (21–23), supporting a role for NADPH oxidase dysfunction in the intestinal inflammation of IBD. It has been reported that p40*^phox^*^−^*^/^*^−^ mice showed increased susceptibility to both DSS- and anti-CD40-induced colitis (19). However, the role of p40*^phox^* in the regulation of intestinal immune response during infectious colitis and the importance of p40*^phox^* expression by phagocytes in controlling and regulating intestinal mucosal bacterial infection is less well understood.

Macrophages and neutrophils contribute significantly to the effector phase of the immune response, i.e., elimination of bacteria, and are also thought to be critical mediators of many chronic inflammatory diseases. These phagocytic cells have evolved a repertoire of antimicrobial mechanisms based on the formation of toxic radicals, including NADPH phagocytic oxidase and inducible nitric oxide synthase. In line with many other investigations, our previous studies showed that macrophages can kill infected bacterial pathogens, such as *Salmonella* and *Citrobacter rodentium via* autophagy (24), a catabolic process that several lines of evidence have suggested can be regulated by ROS and reactive nitrogen species (RNS) *via* undefined molecular mechanisms (25). The generation of ROS has also been shown to induce the activation of inflammasome (26), a cytosolic protein complex that senses microbial stimuli and regulates the maturation of inflammatory cytokine IL-1β production (27). However, mononuclear phagocytes from CGD patients (deficient in ROS production) have been shown to have an increased IL-1β secretion (28). In this study, we utilized genetically engineered mice to determine the role of p40*^phox^* in host innate defense against both systemic and mucosal infection of *S*. Typhimurium, as well as the mechanism of action.

## Materials and Methods

### Mice

Six- to eight-week-old female C57BL/6 [wild-type (WT)] mice were obtained from the Jackson Laboratory (Bar Harbor, ME, USA). p40*^phox^* knockout mice, which have been backcrossed to the B6 background for 10 generations, were kindly provided by Dr. Ramnik Xavier (Massachusetts General Hospital, Boston, MA, USA) (19). All mice were fed autoclaved food and water and maintained in a specific-pathogen-free facility at Massachusetts General Hospital.

### *S*. Typhimurium Infection

Mice were infected orally with 3 × 10^8^ CFU of the streptomycin-resistant SL1344 strain of *S*. Typhimurium. After 72 h of infection, mice were euthanized by CO_2_ inhalation. Spleens and livers were harvested to determine bacterial translocation and systemic inflammatory response. To study whether p40*^phox^* deficiency affects the early mucosal innate immune and inflammatory response to *Salmonella* infection, the streptomycin-pretreatment mouse model was used as described previously (4). Briefly, mice were each given 20 mg of streptomycin, followed 24 h later by oral infection with 10^8^ CFU of *S*. Typhimurium. Ceca were collected for histopathology 24 h after infection. To determine the impact of p40*^phox^* deficiency on the host during *Salmonella* infection, WT mice and mice lacking p40*^phox^* were infected orally with 3 × 10^8^ CFU of *S*. Typhimurium. Body weight loss and survival of mice were monitored daily. Mice were humanely euthanized if they exhibited greater than 15% weight loss.

### Determination of *Salmonella* Translocation

Spleens and livers collected from *Salmonella*-infected mice (WT and p40*^phox^*^−^*^/^*^−^ mice) (with or without streptomycin treatment), were excised using sterile procedure, weighed, homogenized, and plated on Luria–Bertani plates containing 50 µg/ml streptomycin (3). The CFU were quantified. To examine the appearance and distribution of *Salmonella* in cecum and spleen, cryostat sections of the intestinal tissues and spleens were incubated with a rabbit antibody against *Salmonella* (Thermo Fisher Scientific), followed by fluorescein isothiocyanate (FITC) or Cy3-conjugated goat anti-rabbit IgG antibody (Biosource Cat. #554020), and analyzed by immunofluorescence microscopy.

### Histopathology

At necropsy, tissue samples of spleen, liver, and cecum were collected, frozen in Tissue-Tek OCT compound (Miles Inc., Elkhart, IN, USA) and then stored at −80°C. Then, 5-µm sections were cut and stained with hematoxylin and eosin (H&E). Pathology was scored using a modified histological scoring systems previously published in the literature (5, 29, 30). The scores were assessed by determination of infiltration of inflammatory cells, with scores ranging from 0 to 4 (0, normal cell pattern; 1, scattered inflammatory cells in the lamina propria; 2, increased numbers of inflammatory cells in the lamina propria; 3, confluence of inflammatory cells extending into the submucosa; and 4, transmural extension of the infiltrative inflammatory cells), together with the evaluation of cecal tissue damage (score range, 0–4; 0, normal tissue pattern; 1, minimal inflammation and crypt hyperplasia; 2, mild crypt hyperplasia with or without focal invasion of epithelium; 3, obvious crypt hyperplasia, invasion of epithelium, and goblet cell depletion; and 4, extensive mucosal damage and extension through deeper structures of the bowel wall). Stained sections were analyzed without prior knowledge of the type of treatment.

### Immunofluorescence Microscopy

Tissue cryosections were fixed in ice-cold acetone, washed, and then blocked with avidin/biotin agent (Vector Laboratories, Burlingame, CA, USA). To analyze the location and abundance of macrophages and neutrophils, cecal slides were stained with FITC-labeled anti-mouse F4/80 (eBioscience Cat. #11-4801-81) and Cy5-labeled anti-mouse Ly6G (Biolegend Cat. #108410). DNA was stained and mounted using the 4′, 6-diamidino-2-phenylindole (DAPI) (Vector Laboratories). To examine the bacterial killing capacity, peritoneal macrophages were collected from uninfected WT and p40*^phox^* KO mice, grown on cover slips, and infected with *S*. Typhimurium for 1 h (cell/bacteria, 1:10). Cells were then stained with phycoerythrin (PE)-conjugated anti-F4/80 at both 2 and 6 h after infection (24). Internalization and elimination of bacteria by macrophages were visualized by detecting *Salmonella* with a rabbit antibody against *Salmonella*, followed by FITC-labeled anti-rabbit IgG antibody.

### Quantitative Real-time PCR Analysis

Total RNA was isolated from spleen, liver, cecum, and peritoneal macrophages using TRIzol reagent (Invitrogen Life Technologies, Carlsbad, CA, USA) or RNeasy Kit (Qiagen, Valencia, CA, USA) following the manufacturer’s instruction. All RNA samples were reverse transcribed into cDNA using the Superscript First-Strand Synthesis System (Invitrogen Life Technologies). The cDNA samples were then tested for the expression of TNF-α, IL-1β, KC, MCP1, and MIP2 by real-time RT-PCR performed as previously described (29). Results were normalized to GAPDH expression and relative quantification was calculated using the 2^−ΔΔCT^ method.

### Measurement of Cytokine Production by ELISA

Liver and spleen homogenate was prepared for cytokines measurement through ELISA. Protein concentrations were determined using a bicinchoninic acid protein assay. IL-1β was measured by DuoSet ELISA kit from R&D Systems (Cat. #DY401) according to the manufacturer’s instruction. ELISA capture antibodies and biotinylated secondary antibodies for IFN-γ (BD Cat. #554410; 551216), TNF-α (BD Cat. #554416; 557432), and IL-17A (BD Cat. #555068; 555067) were purchased from DB Bioscience. Standard curves were obtained using recombinant murine IFN-γ (BD Cat. #554587), TNF-α (BD Cat. #554589), and IL-17A (eBioscience Cat. #14-8171-80) from BD Bioscience and eBioscience.

### Flow Cytometry Analysis

Splenic single-cell suspensions were prepared from uninfected mice and mice infected orally with 3 × 10^8^ CFU *S*. Typhimurium at 24 h after bacterial infection. Cells were surface stained with fluorescent-conjugated antibodies against CD11b (eBioscience, Cat. #11-0112-85; BD Cat. #553311), Ly6G (BioLegend, Cat. #108410), and F4/80 (eBioscience, Cat. #12-4801-80) before being subjected to flow cytometry analysis with an Accuri C6 FACS machine. Gates set on forward and side angle light scatter were used to exclude dead cells and debris.

### Lucigenin-Dependent Chemiluminescence Assay and 2′,7′-Dichlorofluorescin Diacetate (DCFDA) Intracellular ROS Detection Assay

Reactive oxygen species generation was measured using lucigenin-dependent chemiluminescence. Peritoneal macrophages were collected by washing the peritoneal cavity of WT or p40*^phox^*^−^*^/^*^−^mice with ice-cold PBS. Neutrophils were purified using EasySep™ Neutrophil Enrichment Kit (Stemcell Technologies, Catalog #19762) according to the manufacturer’s instructions. Briefly, mouse bone marrow cells were flushed from femurs and tibias, centrifuged and, after hypotonic lysis of erythrocytes, mixed with biotinylated antibodies and magnetic particles. Neutrophils were then separated by immunomagnetic negative selection. Macrophages and neutrophils were then resuspended in 100 µM lucigenin (5 × 10^7^/ml) and incubated for 15 min on ice. Subsequently, cells were seeded in a black 96-well plate (4 × 10^6^/well) and stimulated with 200 nM PMA (Sigma-Aldrich). Macrophages without PMA treatment were used as a control. Light emission was measured on a TopCount NXT scintillation and luminescence reader (PerkinElmer, Waltham, MA, USA) every 5 min for a period of 1 h at 37°C. Data were expressed as relative light units (RLU) normalized to cell number and corrected for a sample blank. Each experiment was performed in triplicate.

In addition, intracellular ROS generation was measured using DCFDA Cellular Reactive Oxygen Species Detection Assay Kit (Abcam, Ab113851) in accordance with the manufacturer’s instructions and published methods (31). DCFDA is a fluorogenic dye that measures ROS activity within the cell. For these experiments, both intestinal and peritoneal macrophages were isolated from C57BL/6 and p40*^phox^*^−^*^/^*^−^ mice. The macrophages from intestine were isolated using published methods (32). Briefly, small and large intestine were collected and Peyer’s patches were removed. The intestinal tissues were cut open longitudinally (to 1.5 cm pieces) and washed in PBS. The epithelial layer was removed by shaking the tissues in EDTA-containing HBSS/FBS buffer (250 rpm for 20 min at 37°C). The intestinal tissue was then digested with CollagenaseVIII/DNAse I solution and filtered through a 100 µm cell strainer. After washing, the cell pellets were collected. Intestinal macrophages were enriched using magnetic beads (anti-F4/80 and CD11b). The cells were then stained in the diluted DCFDA solution at 37°C for 30 min in the dark, then washed. Cells were seeded in a dark side, clear bottom 96-well microplate (2 × 10^5^ stained cells/well). The cells were then stimulated with 20 nM PMA (Sigma-Aldrich) or infected with *S*. Typhimurium (cell/bacteria, 1:100). Macrophages without PMA treatment or *Salmonella* infection were used as a control. The microplates were read using a fluorescent plate reader (FluoSTAR OPTIMA-BMG Labtech) with the excitation wavelength set at 485 nm and emission wavelength at 535 nm. Results are expressed as fluorescent intensity of samples minus blank (DCFDA-unstained cells). Each experiment was performed in triplicate.

### Gentamicin Protection Assay

The resident peritoneal macrophages were collected from uninfected WT and p40*^phox^* knockout mice. After 2 h of incubation in complete DMEM, non-adherent cells were removed by washing. The adherent cells were cultured at 37°C overnight and then exposed to 10^7^ *S*. Typhimurium (cell/bacteria, 1:10) for 1 h in antibiotic-free medium. After completion of the infection period, cells were washed with PBS and then incubated with gentamicin-containing medium (100 µg/ml) for 2 h to eliminate extracellular bacteria. Cells were then lysed immediately in sterile 1% Triton X-100 in water or, after a further 4 h, in medium containing 10 µg/ml gentamicin. Serial dilutions of the cell lysates were plated on LB agar containing streptomycin overnight. Colonies were counted after incubation and bacterial numbers present inside the cells at each time point were calculated (24, 33).

### Statistical Analysis

Results are expressed as the mean ± SEM. Survival curves of infected mice (WT and p40*^phox^*^−^*^/^*^−^) were compared using Kaplan–Meier analysis followed by log-rank test. Statistical differences were determined using one-way ANOVA with Tukey’s *post hoc* test, the two-tailed Student’s *t*-test or paired *t*-test (body weight loss) with GraphPad Prism (GraphPad Software, San Diego, CA, USA). Significance was defined as a *P*-value <0.05.

## Results

### p40^*phox*^ Deficiency Increases Morbidity of Mice during *Salmonella* Infection

**Figure 1:**
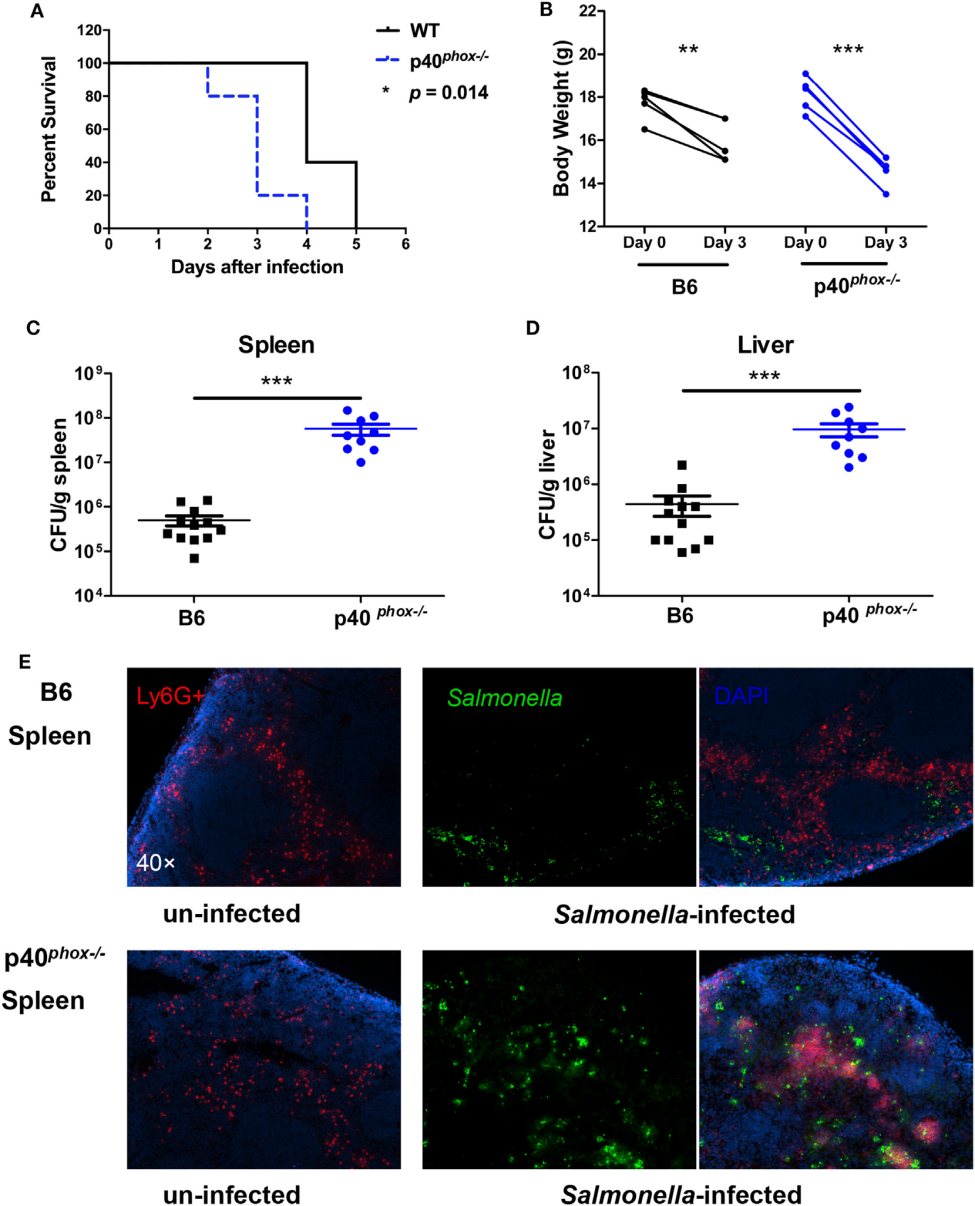
p40*^phox^* deficiency exacerbates bacterial translocation and mortality in *Salmonella*-infected mice. Wild-type (WT) mice and mice lacking p40*^phox^* were infected orally with 3 × 10^8^ CFU of *Salmonella enterica* serovar Typhimurium (SL1344 strain). **(A)** Mice were monitored daily for survival. **(B)** Body weight changes were measured 72 h post infection. Significance was determined using a paired *t*-test for body weight (*n* = 5/group; *p < 0.05; **p < 0.01; ***p < 0.001). Bacterial translocation to spleens **(C)** and livers **(D)** was determined 72 h after infection using a Student’s *t*-test. The number of CFUs per gram of tissue is shown (*n* = 9–12/group; ***p < 0.001). **(E)** Immunofluorescence microscopy data show the distribution of *Salmonella* (in Green) in spleen sections (magnification 40×). Neutrophils were identified by positive staining with anti-Ly6G. Data shown are generated from three independent experiments.

NADPH oxidase is critical in host defense against invading pathogens (34). To investigate the role of p40*^phox^*, one of the subunits of the NADPH oxidase, in host protective and inflammatory responses against *Salmonella* infection and in the regulation of the severity of bacterial-induced disease, p40*^phox^*^−^*^/^*^−^ mice and C57BL/6 WT control mice were infected orally with 3 × 10^8^ CFU of *S*. Typhimurium (SL1344 strain). As expected, mice infected with *S*. Typhimurium exhibited significant body weight loss and high mortality (100%) by day 5 of infection in WT mice. By contrast, p40*^phox^*^−^*^/^*^−^ mice developed more severe disease, resulting in a more significant body weight loss and early mortality reaching 100% mortality by 4 days after inoculation (Figures 1A,B). To examine whether the increased susceptibility of p40*^phox^*-deficient mice to *Salmonella* infection was related to poor control of bacterial replication, bacterial translocation to spleens and livers was determined 72 h after infection. Significantly, greater bacterial numbers were present in the spleens (Figure 1D) and livers (Figure 1C) of p40*^phox^*^−^*^/^*^−^ mice compared with C57BL/6 controls, suggesting an impaired mucosal barrier function and inability to effectively clear microbes in p40*^phox^*-deficient mice. This is further supported by the results from our immunofluorescence microscopic analysis revealing enhanced bacterial dissemination in the spleen tissues of p40*^phox^*-deficient mice (Figure 1E).

**Figure 2:**
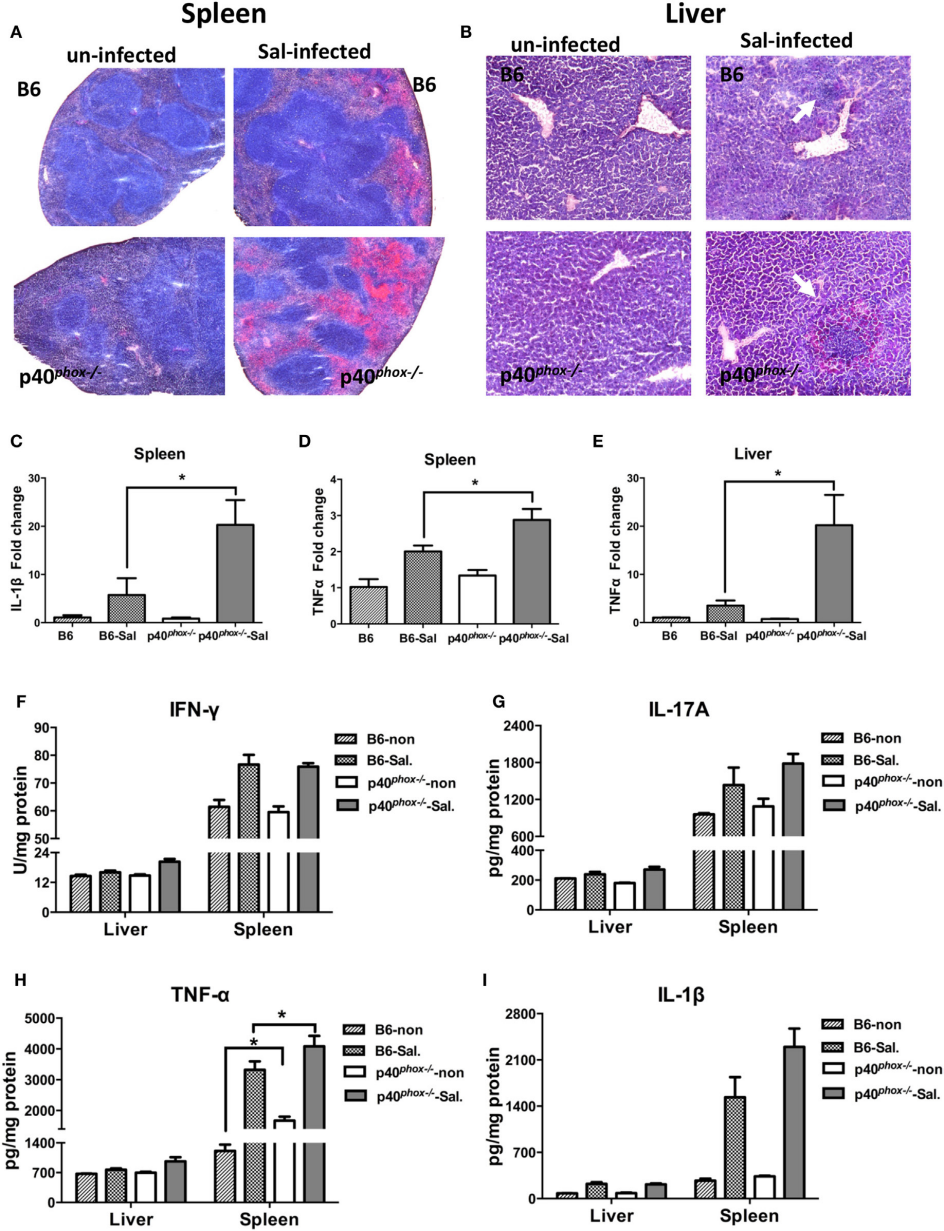
p40*^phox−/−^* mice develop more severe spleen and liver inflammation after *Salmonella enterica* serovar Typhimurium (*S*. Typhimurium) infection. Spleens and livers were harvested 72 h after *S*. Typhimurium infection. Representative spleen **(A)** and liver **(B)** sections stained by hematoxylin and eosin [**(A)** magnification 20×; **(B)** magnification 40×]. Arrows indicate inflammatory infiltrates. **(C–E)** The gene expression of IL-1β and TNF-α was assessed *via* quantitative PCR. Values represent the fold increase compared to baseline obtained from uninfected wild-type (WT) mice (*n* = 5/group; *p < 0.05). Significance was determined by one-way ANOVA with Tukey’s *post hoc* test. **(F–I)** Cytokine levels of IFN-γ, IL-17A, TNF-α, and IL-1β were measured by ELISA. The data shown are mean ± SEM from one of three experiments performed showing similar results (*n* = 5/group; *p < 0.05). Significance was determined by a Student’s *t*-test.

The increased tissue bacterial loads were accompanied by exacerbated inflammation and tissue damage, which was evidenced by the observations from analysis of histopathology and pro-inflammatory cytokine profiles of these organs from WT and p40*^phox^*^−^*^/^*^−^ mice with and without *S*. Typhimurium infection. Histological analysis of infected mice revealed extensive pathological changes in the spleen, evidenced by a neutrophilic infiltrate and congestion within the red pulp in *Salmonella*-infected WT mice (Figure 2A). In contrast to WT mice, p40*^phox^* knockout mice developed more severe tissue damage, characterized by red pulp congestion, thrombosis, and splenic architecture disruption (Figure 2A). Histological analysis of the liver sections from *Salmonella*-infected mice showed massive necrosis accompanied by a mixed inflammatory cell infiltration in both WT and p40*^phox^*-deficient mice. However, more pronounced and extensive hepatic lesions were noted in *Salmonella*-infected p40*^phox^*^−^*^/^*^−^ mice compared to WT mice (Figure 2B). These results suggest that *Salmonella* infection of p40*^phox^* KO mice results in an exacerbated inflammation and tissue injury in the spleen and livers due to the higher numbers of bacteria in the tissues.

Consistent with more severe inflammatory changes in the spleen and liver, we found there was a significant upregulation of gene expression of inflammatory cytokine IL-1β (Figure 2C) and TNF-α (Figures 2D,E) in infected p40*^phox^* KO mice. Furthermore, ELISA analysis of cytokine concentration in spleen and liver homogenates confirmed that *Salmonella* infection induced a marked increase in production of TNF-α in spleen from mice lacking p40*^phox^* (Figure 2H). Moreover, a trend of increase was found for IL-1β production in spleen from p40*^phox^*^−^*^/^*^−^ mice (Figure 2I) while there was no statistical significance. However, the levels of IFN-γ (Figure 2F) and IL-17A (Figure 2G) present in the livers and spleens of infected p40*^phox^*^−^*^/^*^−^ mice were comparable to WT levels. These findings suggest that p40*^phox^* deficiency results in increased pro-inflammatory cytokine production (including TNF-α and IL-1β) during bacterial infection, which may be responsible for the observed enhanced tissue damage. Collectively, the data demonstrate that p40*^phox^* is critical for host resistance and survival during *Salmonella* infection.

### p40^*phox*^ Deficiency in *S*. Typhimurium-Infected Mice Enhances the Recruitment of Macrophages

**Figure 3:**
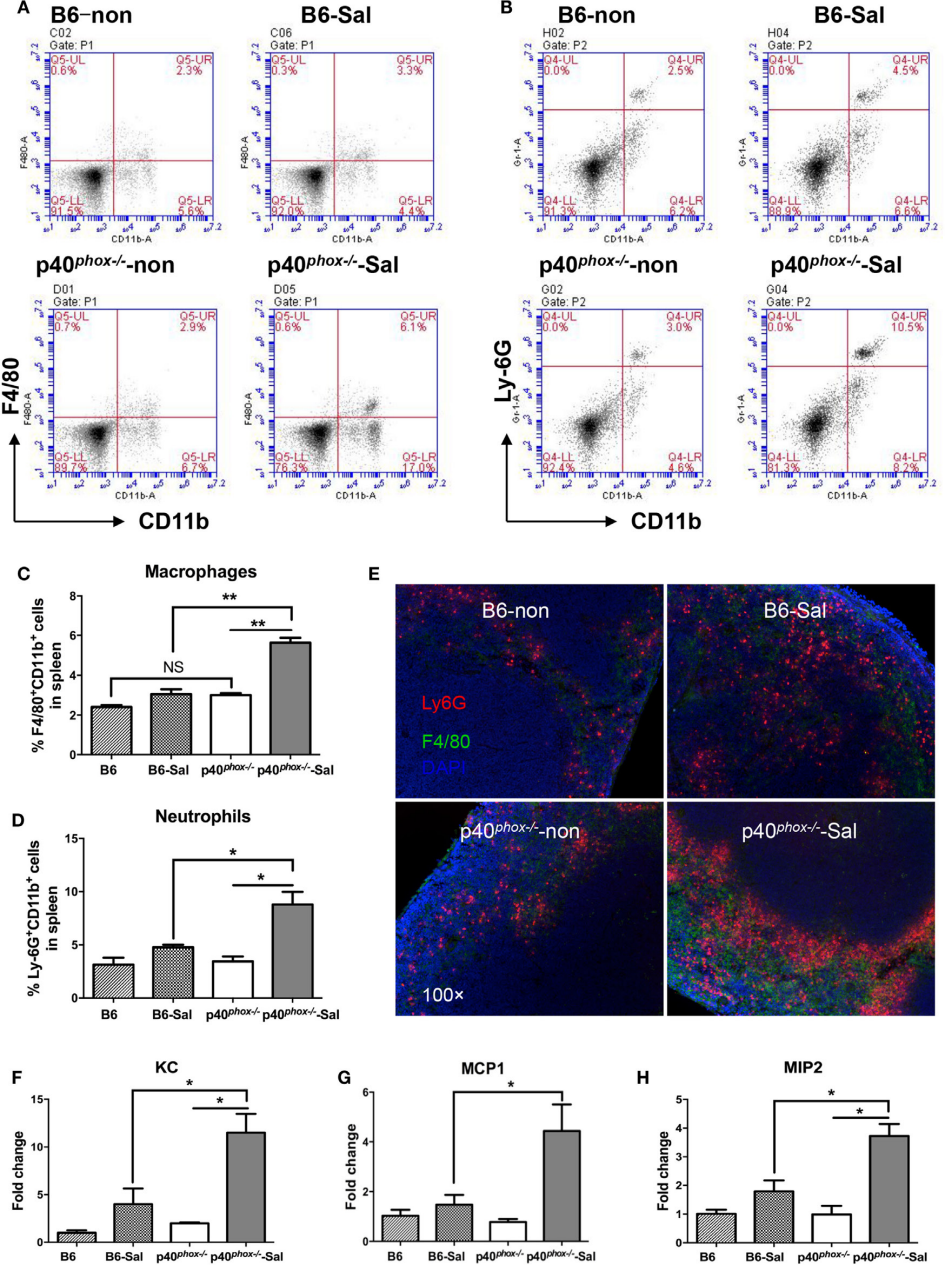
p40*^phox^* deficiency in *Salmonella enterica* serovar Typhimurium (*S*. Typhimurium)-infected mice enhances the recruitment of macrophages. Mice were infected with *S*. Typhimurium (3 × 10^8^ CFU) and sacrificed 24 h post infection. Single-cell suspensions from spleens were prepared and stained for macrophages with anti-F4/80 and anti-CD11b **(A)** or neutrophils with anti-Ly6G and anti-CD11b antibodies **(B)**. The percentages of F4/80^+^CD11b^+^ macrophages **(C)** and Ly6G^+^CD11b^+^neutrophils **(D)** are shown as mean ± SEM (*n* = 5/group; NS = Not Significant; *p < 0.05; **p < 0.01). FACS plots are representative of three independent experiments. Spleen from uninfected B6 and p40*^phox^* KO mice (left) and infected B6 and p40*^phox^* KO mice (right) were stained with anti-F4/80 for macrophages and anti-Ly6G (Red) for neutrophils (magnification × 100) **(E)**. **(F–H)** Analysis of splenic chemoattractants (KC, MCP1, and MIP2) gene expression was performed by quantitative PCR (*n* = 5/group; *p < 0.05). Significance was determined by one-way ANOVA with Tukey’s *post hoc* test.

To further study the influence of p40*^phox^* deficiency on host innate immunity against *Salmonella* infection, we next analyzed the frequency of macrophages and neutrophils in the splenic tissue of p40*^phox^* knockout mice with *Salmonella* infection. Our FACS analysis of spleen cells revealed that the frequency of macrophages expressing CD11b^+^ F4/80^+^ was significantly enhanced in infected p40*^phox^*^−^*^/^*^−^ mice compared to WT mice (Figures 3A,C). A significant increase in splenic Ly6G^+^CD11b^+^ neutrophil numbers was also observed between WT mice and p40*^phox^*-deficient mice with *Salmonella* infection (Figures 3B,D). These results are further supported by the results from our immunofluorescence microscopic analysis of spleen revealing enhanced macrophages and neutrophil in the spleen tissues of p40*^phox^*-deficient mice (Figure 3E). These data suggest that loss of p40*^phox^* results in increased transmigration of macrophages and neutrophils to the site of infection. We next determined the role for p40*^phox^* in the regulation of bacteria-induced chemoattractant expression, which regulates macrophage and neutrophil recruitment. Accordingly, we assessed the expression of the three major chemoattractants: KC (CXCL1), MCP1 (CCL2), and MIP2 (CXCL2) in the spleen tissues. Our RT-PCR results showed that *Salmonella* infection induced an upregulation of KC, MCP1, and MIP2 gene expression in the spleens. This bacteria-induced upregulation of chemokines expression was more pronounced in the spleen of p40*^phox^*-deficient mice than that detected in WT mice (Figures 3F–H). These observations show that p40*^phox^* deficiency in mice may enhance the recruitment of phagocytes to the infection site by regulating the expression of KC, MCP1, and MIP2 during *Salmonella* infection and suggest that the excessive accumulation of granulocytes in the spleens of infected p40*^phox^* KO mice may thereby enhance the inflammatory response.

### p40^*phox*^ Deficiency Exacerbates *Salmonella*-Induced Mucosal Injury

**Figure 4:**
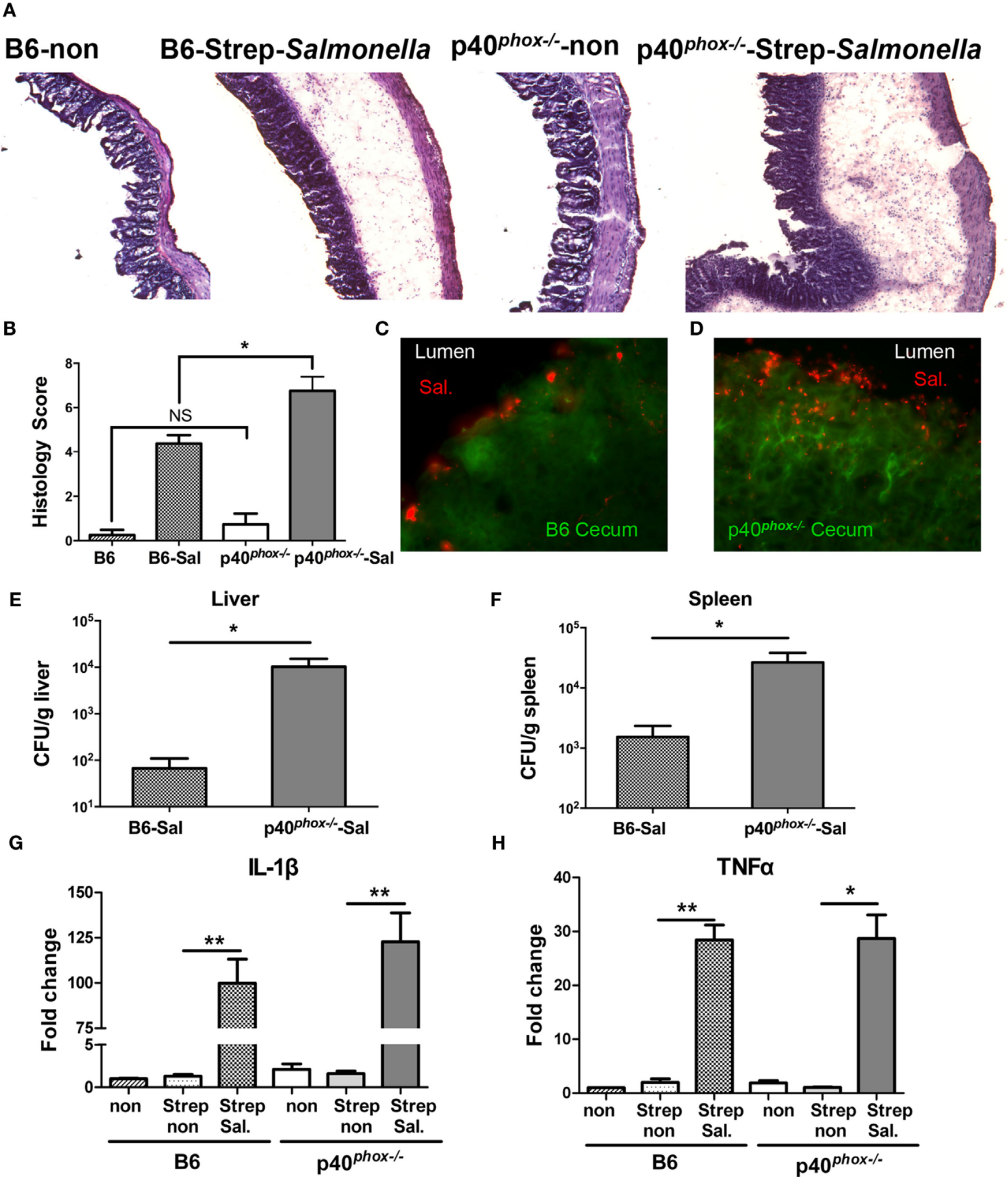
p40*^phox−/−^* mice show more severe *Salmonella*-induced mucosal injury and enterocolitis. Mice lacking p40*^phox^* and wild-type (WT) mice were treated with streptomycin prior to infection with *Salmonella enterica* serovar Typhimurium (10^8^ CFU) and sacrificed 24 h post infection. **(A)** Representative hematoxylin and eosin-stained cecum sections from each group of mice (original magnification 40×). **(B)** Histopathological score of cecal inflammation in mice with or without infection of *Salmonella*. The scores were assessed by determination of infiltration of inflammatory cells (score range, 0–4), together with the evaluation of cecal tissue damage (score range, 0–4). **(C,D)** Representative immunofluorescence-stained cecum sections from each group of mice showing increased intestinal bacterial loads in the cecum of p40^phox^ deficient mice compared to WT mice. Numbers of bacteria recovered from liver **(E)** and spleen **(F)** homogenates of *Salmonella*-infected mice were determined using a Student’s *t*-test. The number of CFUs per gram of tissue is shown (*n* = 5–6/group; *p < 0.05). Cecal levels of IL-1β **(G)** and TNF-α **(H)** were determined by quantitative PCR (*n* = 5/group; *p < 0.05; **p < 0.01). Data are representative of two independent experiments.

Our data mentioned above suggest that *Salmonella* induced more severe systemic infection (i.e., spleen and liver infection) in p40*^phox^*-deficient mice. To study whether and how p40*^phox^* deficiency affects the early mucosal innate immune and inflammatory responses, we utilized the streptomycin-pretreatment model of *Salmonella* infection, which has been widely used in the field to analyze *Salmonella*-induced intestinal inflammation (2, 4, 5, 29). In brief, mice were treated with streptomycin prior to infection with *S*. Typhimurium (10^8^ CFU) and sacrificed 24 h post infection. Our results showed that mice infected with *Salmonella* developed acute disease, as indicated by a hunched posture, disturbed body hair, soft stool, and body weight loss soon after bacterial inoculation. A more severe disease status was observed in p40*^phox^*^−^*^/^*^−^ mice after *Salmonella* infection (data not shown). Histological examination of cecum showed extensive pathological changes that were typical in this model, including pronounced submucosal edema, dramatic disruption of epithelial architecture, and marked inflammatory cell infiltrates (Figure 4A). The ceca of p40*^phox^*^−^*^/^*^−^ mice showed a more pronounced edema of the intestinal tissue and increased inflammatory cellular infiltration (Figure 4A) than that detected in WT mice. These cecal inflammatory features were absent from uninfected mice (Figure 4A). Using the pathological scoring system (29), we found that the cecal inflammation was notably more severe in p40*^phox^* KO mice than in WT mice (Figure 4B). Immunofluorescence microscopic analysis of infected intestine (cecum) revealed increased bacterial loads in the tissue of the p40*^phox^* KO mice (Figure 4D) compared to WT B6 mice (Figure 4C). Further analysis of tissue bacterial loads showed that the enhanced severity of intestinal injury in the p40*^phox^* KO mice was associated with significantly higher bacterial numbers in livers (Figure 4E) and spleens (Figure 4F) compared to WT mice, again an indication of more severely impaired bacterial killing activity in p40*^phox^*-deficient mice. Moreover, and consistent with the severe intestinal injury, the mRNA expression levels of IL-1β (Figure 4G) and TNF-α (Figure 4H) were upregulated markedly in the cecum of infected mice compared with uninfected mice at 24 h post exposure to *Salmonella*.

**Figure 5:**
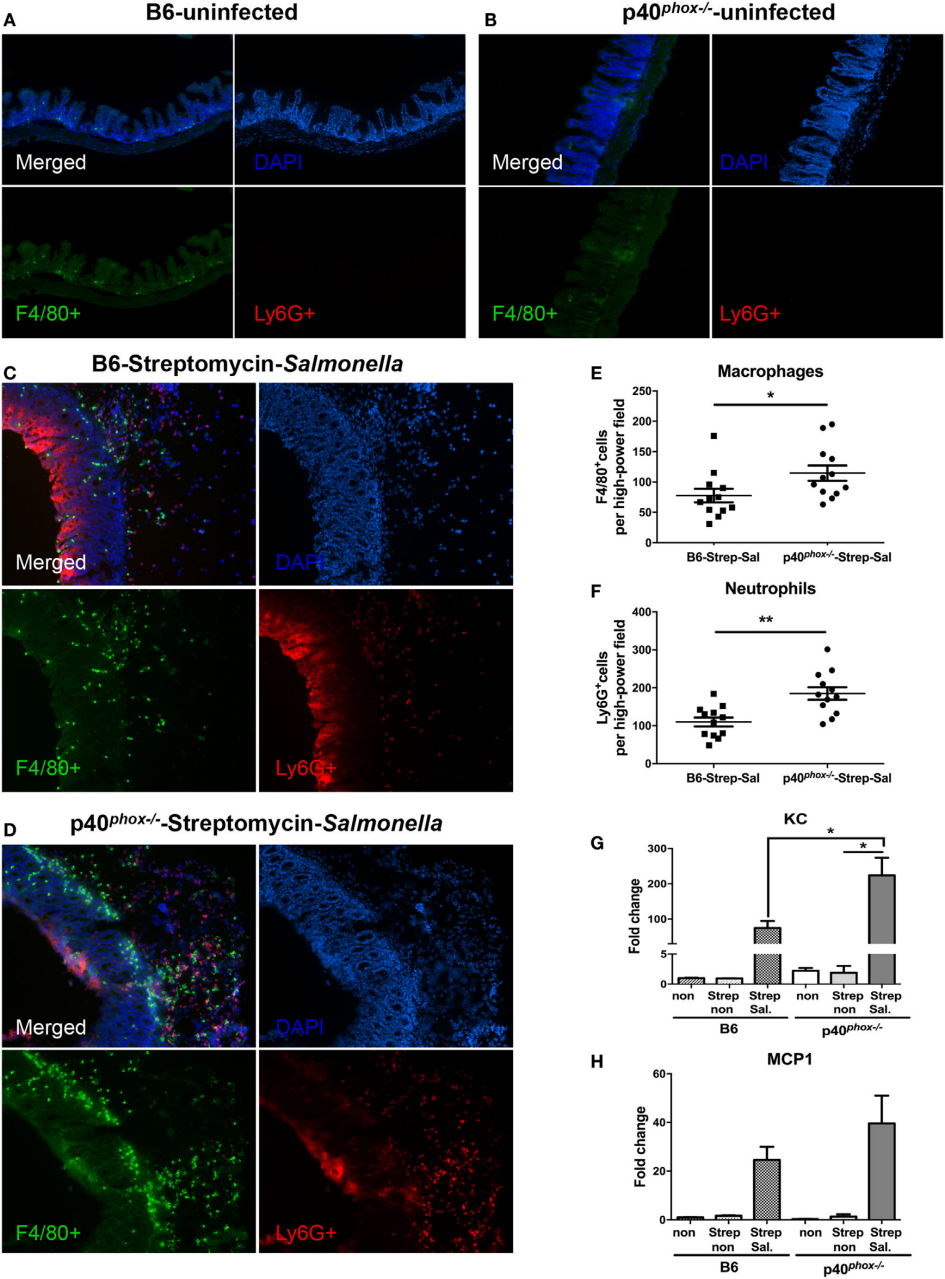
p40*^phox^* deficiency enhances the recruitment of macrophages and neutrophils in cecal lamina propria. Mice deficient in p40*^phox^* and normal mice were treated with streptomycin prior to *Salmonella enterica* serovar Typhimurium infection and sacrificed 24 h post infection. Ceca from uninfected B6 **(A)** and p40*^phox^* KO **(B)** mice and infected B6 **(C)** and p40*^phox^* KO **(D)** mice were stained with anti-F4/80 for macrophages and anti-Ly6G (Red) for neutrophils (magnification 100×). The mean number of F4/80^+^ cells **(E)** and Ly6G^+^ cells **(F)** detected in each high power field was determined by counting four fields from each sample (samples from three mice per group were counted) (*p < 0.05; **p < 0.01). Cecal levels of KC **(G)** and MCP1 **(H)** were determined by quantitative PCR (*n* = 5/group; *p < 0.05). Data are representative of two independent experiments.

Next, we examined whether p40*^phox^* deficiency affected the infiltrating cell population in cecal lamina propria of mice in the streptomycin-pretreated *Salmonella*-colitis model using the immunofluorescence approach. Ceca sections were stained with anti-F4/80 (35) for macrophages and anti-Ly6G for neutrophils (29). As shown in Figures 5C,D, the immunofluorescence examination revealed a clear infiltration of F4/80^+^ macrophages and Ly6G^+^ neutrophils in the cecal submucosa in the infected mice, but was absent in the ceca of the uninfected mice in both groups (Figures 5A,B). A significantly higher frequency of both F4/80^+^ macrophages and Ly6G^+^ neutrophils was noted in the cecal lamina propria of p40*^phox^* knockout mice with *Salmonella* infection (Figures 5E,F) compared to WT mice. The observed increased macrophage and neutrophil infiltration in the intestinal tissue was found to be correlated with upregulation of KC (Figure 5G) and MCP1 (Figure 5H) in the cecum of infected mice compared with uninfected mice at 24 h post exposure to *Salmonella*. Notably, a much higher expression level of KC was observed in the inflamed cecum of p40*^phox^*^−^*^/^*^−^ mice than in WT mice (Figure 5G). Taken together, these results suggest that p40*^phox^*^−^*^/^*^−^ mice develop exacerbated *Salmonella*-induced enterocolitis, characterized by severe tissue injury and enhanced macrophages and neutrophil infiltration in the intestinal tissue.

### p40^*phox*−*/*−^ Macrophages Exhibit Normal Bacterial Phagocytosis and Reduced Bactericidal Activity

**Figure 6:**
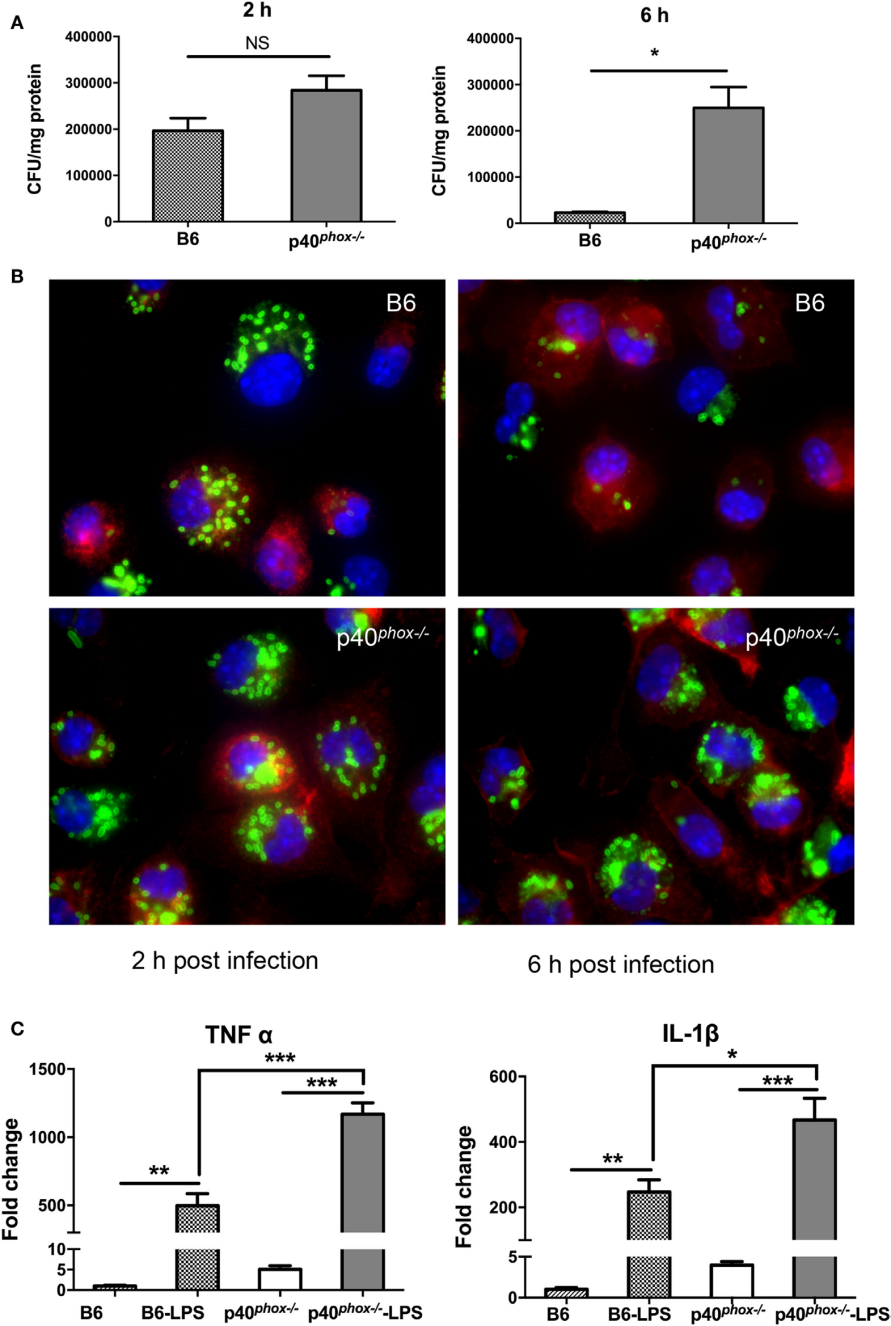
p40*^phox^*^−^*^/^*^−^ macrophages exhibit normal bacterial phagocytosis and reduced bactericidal activity. Peritoneal macrophages were isolated from wild-type (WT) and p40*^phox^*^−^*^/^*^−^mice. **(A)** Peritoneal macrophages were exposed to *Salmonella enterica* serovar Typhimurium (10 bacteria/cell) for 1 h. At different time points after infection (2, 6 h), the number of intracellular bacteria was determined by plating cell lysates onto LB plates supplemented with streptomycin. Results are expressed as mean ± SEM (*n* = 5/group; NS = Not Significant; *p < 0.05). The data shown are representative of three experiments with similar results. **(B)** Immunofluorescence microscopy data show the number of internalized *Salmonella* in peritoneal macrophages (stained with Cy3 anti-F4/80) at 2 and 6 h after gentamicin treatment. *Salmonella* bacteria were detected with a rabbit antibody against *Salmonella*, followed by fluorescein isothiocyanate (FITC)-labeled anti-rabbit IgG antibody. **(C)** Real-time PCR analysis reveals that *Salmonella* LPS treatment (100 ng/ml) significantly upregulated the gene expression of IL-1β and TNF-α in both WT and p40*^phox^*^−^*^/^*^−^ cells (*n* = 3–5/group; *p < 0.05; **p < 0.01; ***p < 0.001). The results are displayed as mean ± SEM and are representative of three independent experiments.

Despite the observed increase in macrophages and neutrophils, our results also showed that p40*^phox^*^−^*^/^*^−^ mice have elevated numbers of bacteria present in infected tissue, suggesting that these phagocytes are unable to effectively control bacterial multiplication in p40*^phox^*^−^*^/^*^−^ mice. Previous study showed that the NADPH oxidase deficiency of p40*^phox^*^−^*^/^*^−^ neutrophils were severely deficient in bacterial killing (20). In this study, we evaluated the bacterial killing capacity of p40*^phox^*^−^*^/^*^−^ macrophages, as macrophages are the major population of tissue-resident mononuclear phagocytes, contributing significantly to the elimination of bacteria, and are the major source of critical mediators of inflammation. We isolated peritoneal macrophages from WT and p40*^phox^*^−^*^/^*^−^ mice, infected the cells with *Salmonella in vitro*, and then used gentamicin protection assay as a measure of macrophage microbicidal function (24). Our results showed that the macrophages isolated from p40*^phox^*^−^*^/^*^−^ mice internalized similar numbers of bacteria at 2 h after infection compared with cells isolated from WT mice (Figure 6A). However, at 6 h after initial bacterial exposure, the number of viable bacteria recovered from p40*^phox^*-deficient macrophages was significantly higher than that in macrophages isolated from WT mice (Figure 6A), indicating an impaired bacterial killing capacity of p40*^phox^*^−^*^/^*^−^ macrophages.

To further determine the functional alterations of p40*^phox^*^−^*^/^*^−^ macrophages, peritoneal macrophages from WT and p40*^phox^*^−^*^/^*^−^ mice were collected, grown on cover slips, and infected with *Salmonella*. After incubation in gentamicin-containing medium, we visualized the influence of p40*^phox^* deficiency on the uptake and subsequent elimination of *Salmonella* by macrophages *in vitro* using immunofluorescent staining approach at 2 and 6 h after bacterial infection. Our results showed that at the early time point (2 h), F4/80^+^ macrophages isolated from WT and p40*^phox^*^−^*^/^*^−^ mice internalized bacteria equally well, evidenced by the detection of similar frequency and intensity of *Salmonella* in macrophages (Figure 6B). At the 6 h time point, the number of cells that contained bacteria was markedly reduced in WT macrophages and compared to that detected in p40*^phox^*^−^*^/^*^−^ macrophages (Figure 6B), confirming that macrophages from p40*^phox^*^−^*^/^*^−^ mice are impaired in killing internalized bacteria.

Our cytokine production analysis of *Salmonella-*infected tissues revealed a more pronounced increase in TNF-α and IL-1β response in p40*^phox^*-deficient mice (Figures 2 and 4). To define the impact of p40*^phox^* deficiency on bacterial antigen-induced macrophage cytokine response, we collected peritoneal macrophages from WT and p40*^phox^*^−^*^/^*^−^ mice and exposed the cells to *Salmonella* LPS *in vitro* (36). Our real-time PCR analysis revealed that LPS treatment significantly upregulated the gene expression of TNF-α and IL-1β in both WT and p40*^phox^*^−^*^/^*^−^ cells, whereas the expression levels of IL-1β and TNF-α in p40*^phox^*^−^*^/^*^−^ macrophages were significantly higher than those isolated from normal mice (Figure 6C). These results suggest that p40*^phox^* deficiency induces dysregulation of pro-inflammatory responses of macrophages.

### p40^*phox*−*/*−^ Macrophages Exhibit Reduced ROS Generation

**Figure 7:**
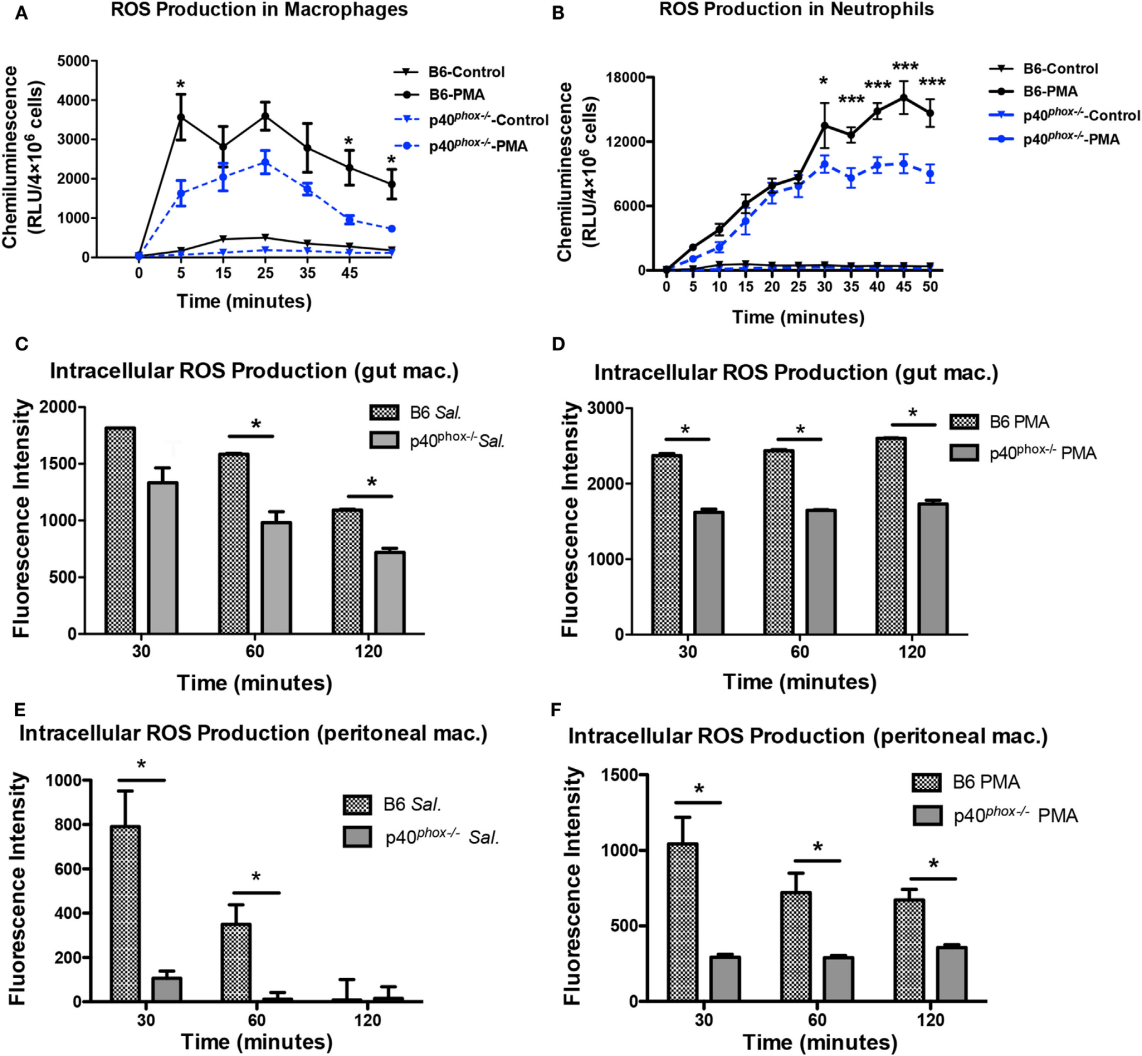
p40*^phox^*^−^*^/^*^−^ macrophages have impaired reactive oxygen species (ROS) production. Intestinal and peritoneal macrophages were isolated from wild-type and p40*^phox^*^−^*^/^*^−^mice. **(A,B)** Time course of lucigenin-elicited chemiluminescence in peritoneal macrophages after addition of 200 nM PMA or the same volume of HBSS. ROS release was monitored every 5 min for a period of 1 h at 37°C. **(C–F)** Bacterial and PMA-induced intestinal **(C,D)** and peritoneal macrophage **(E,F)** intracellular ROS production was measured using the 2′,7′-dichlorofluorescin diacetate (DCFDA) Cellular ROS Detection Assay Kit. Results are expressed as fluorescent intensity of samples minus blank (DCFDA-unstained cells) (*n* = 3–5 mice/group; *p < 0.05). Each experiment was performed in triplicate.

Using a lucigenin-dependent chemiluminescence assay, we measured PMA-induced ROS generation by peritoneal macrophages as well as neutrophils isolated from WT and p40*^phox^*^−^*^/^*^−^ mice and confirmed that the production of ROS was markedly reduced in p40*^phox^*^−^*^/^*^−^ cells (Figures 7A,B). However, PMA-induced ROS production was not completely abolished in p40*^phox^*^−^*^/^*^−^ macrophages and neutrophils. Upon phagocytosis of the pathogens, the ROS are produced. In the next set of experiments, using the DCFDA Cellular ROS Detection Assay Kit, we determined the impact of p40*^phox^* deficiency on the intracellular ROS production of both intestinal (Figures 7C,D) and peritoneal (Figures 7E,F) macrophages in response to *S*. Typhimurium infection. Intestinal and peritoneal macrophages were isolated from both WT and p40*^phox^*-deficient mice and stained in the diluted DCFDA solution before being infected with *S*. Typhimurium or treated with PMA. Our results show that *Salmonella*-induced macrophage ROS production was significantly higher in cells from WT mice compared to p40*^phox^*^−^*^/^*^−^ mice (Figures 7C,E). Moreover, PMA-induced ROS production of macrophages was found to be markedly lower in p40*^phox^*^−^*^/^*^−^ macrophages compared to WT cells (Figures 7D,F), confirming the results from lucigenin-dependent chemiluminescence assay. These results demonstrate that intracellular ROS generation by macrophages from p40*^phox^*^−^*^/^*^−^ mice was significantly impaired.

## Discussion

*Salmonella* infection is a major cause of human food-borne gastroenteritis worldwide. To effectively control the infection, molecules that can enhance the microbicidal capacity of phagocytes, such as phagocyte NADPH oxidase, are required. Individuals who have inherited deficiencies of NADPH oxidase show enhanced susceptibility to infection and develop CGD (16). Mice deficient for *Cybb* gene, encoding gp91*^phox^*, the core component of the NADPH oxidase complex, failed to control infection with a normally avirulent bacterial infection (6). In this study, we determined the functional role of p40*^phox^* in the effective control of both systemic and mucosal infection of *Salmonella*. Our results showed that defects in p40*^phox^* in mice resulted in significantly increased morbidity and mortality during *Salmonella* infection. This is evidenced by the detection of higher tissue bacterial loads in the liver and spleen, supporting the role for p40*^phox^* in restricting the penetration and growth of invading bacteria in intestinal mucosa, and enhanced level of bacteria-induced pro-inflammatory cytokine response. Moreover, we observed that p40*^phox^* deficiency led to markedly increased recruitment of F4/80^+^ macrophages and Ly6G^+^ neutrophils in the infected tissues. Functional analysis of macrophages revealed that, in addition to the anticipated reduced ROS generation, the bacterial killing capacity of macrophages was significantly impaired in p40*^phox^*-deficient mice and that p40*^phox^*-deficient macrophages produced significantly more IL-1β and TNF-α. These findings indicate that the p40*^phox^* of NADPH oxidase plays an essential role in suppressing intracellular multiplication of *Salmonella* in macrophages and in the regulation of macrophage inflammatory response to bacterial infection.

Our results showed that p40*^phox^* deficiency in mice significantly increased the bacteria-induced infiltration of macrophages and neutrophils into spleen and ceca mucosa (Figures 3 and 5), which correlated with an upregulation of expression of KC and MIP2 in the infected tissues, and yet, higher bacterial loads were detected in these tissues. In line with previous report showing that p40*^phox^* deficiency in mice resulted in impaired neutrophil function (20), our functional analysis of macrophages from p40*^phox^*-deficient mice demonstrated functional defect of macrophages. This is evidenced by the results showing the elimination of *Salmonella* is significantly compromised in these cells. The uncontrolled bacterial infection and elevated bacterial loads may contribute to the observed enhanced pro-inflammatory cytokine production and exacerbated *Salmonella*-induced mucosal inflammation. This observation is in line with the observations in CGD patients, who have impaired ROS production and increased bacterial and fungal infections (16).

It was also reported that in the TNBS colitis model CGD mice (Nox2^−^*^/^*^−^) developed an accentuated colitis (37), with exaggerated neutrophil and macrophage infiltration and bacterial dissemination deep into the crypts (37). The observation of increased neutrophils and macrophages in Nox2^−^*^/^*^−^ mice has been suggested due to a functional failure of macrophages in clearance of neutrophils (38), as neutrophil numbers decrease during resolution of TNBS colitis as a result of macrophage clearance (39). New evidence indicated that the infiltration of neutrophils in TNBS colitis consumed sufficient O_2_ to stabilize intestinal epithelial cell hypoxia-inducible factor (HIF), resulting in inflammation resolution. In contrast to WT mice, CGD mice do not provoke mucosal hypoxia and exhibit an increased susceptibility to intestinal inflammation (37). It is clear that further investigation is needed for defining the potential role for p40*^phox^* in the regulation of kinetic and spatial distribution and migration of macrophage and PMN and mucosal integrity in the context of bacterial infection and inflammation.

After invasion of macrophages, *Salmonella* remain inside a membrane-bound compartment, the *Salmonella*-containing vacuole (SCV). We have not directly measured ROS levels inside the SCV, a technically challenging task that requires a special *Salmonella* strain expressing a redox sensitive green fluorescent protein (40). However, the methods that we have used measure total cellular ROS and are widely used in the field. The fact that they clearly demonstrate a p40*^phox^*-dependent decrease in ROS production that correlates with increased intracellular survival of *Salmonella* indicates their functional relevance. How p40*^phox^* deficiency affects ROS levels inside the SCV remains to be determined.

Reactive oxygen species generation *via* NADPH oxidase by innate immune cells and the epithelium plays important role in host defense against infection. Although the current study focused on the role of p40*^phox^* in innate immune cells in host protection against bacterial infection, epithelial cells also produce ROS, which participate in regulating intracellular signals for barrier repair and healing (41). In the GI tract, the main sources of ROS are NADPH oxidase enzymes. There are seven members in the family of ROS-generating NADPH oxidase, including NOX1–5 and two dual oxidases (DUOX1 and DUOX2). NOX2 is referred to as the phagocyte NADPH oxidase because it was first described in neutrophils and macrophages (42) and is a major ROS-producing enzyme with bactericidal activity (43). The main oxidases expressed along the GI tract are NOX1, often called “colon NADPH oxidase” due to the high expression in colon epithelium and DUOX2, which can be detected throughout all of the digestive tract, particularly in cecum and colon epithelium (44). DUOX2 is thought to participate in antimicrobial defense in the host mucosa (45–47). It has been shown that loss of functional DUOX enzymes lead to increased mucosal colonization by *Helicobacter felis*, enhanced shedding of bacterial antigens and virulence factors, and severe gastritis in Duoxa (Dual oxidase maturation factor) deficient mice (45). These results suggest an antimicrobial role for Duox at the apical surface in the mucus layer of the gastric epithelium controls growth of *H. felis* (45). Recently, two functionally altered NOX1 and DUOX2 variants in very early onset (VEO) IBD patients have been identified, both of which are associated with severe pancolitis and reduced ROS production *in vitro* (48, 49), suggesting that reduced epithelial ROS may provoke a pro-inflammatory phenotype similar to that in CGD patients. Interestingly however, a recent investigation using mice with complete or epithelium-restricted deficiency in *Cyba*, encoding the p22*^phox^* component of NADPH oxidase, showed that epithelial deficiency of *Cyba* resulted in protection from *Citrobacter rodentium* infection (50). In that study, it was also shown that intestinal ROS altered the gut microbiota composition, which is evidenced by the detection of enrichment of H_2_O_2_-producing bacterial strains in mice with epithelial *Cyba* deficiency. This study further showed that *C. rodentium* virulence can be attenuated by H_2_O_2_-mediated suppression of the virulence-associated LEE pathogenicity island (50). Although a direct role for p40*^phox^* in regulating intestinal epithelial cell protection against enteric bacterial infection needs to be investigated, these published data indicate the possibility that reduced intestinal epithelial ROS generation will influence mucosal responses to both commensal and pathogens, the pathogenesis of IBD and intestinal inflammation.

In both systemic and mucosal infection models utilized in the current study, hyperproduction of IL-1β was detected in the infected tissues in p40*^phox^*-deficient mice (Figures 2 and 4). IL-1β is produced as a biologically inactive precursor and the caspase-1-dependent IL-1β secretion requires the activation of inflammasome, a cytosolic protein complex that senses microbial and endogenous stimuli, which is implicated in host protection and a variety of inflammatory diseases (51). Evidence also showed that PMN cells isolated from CGD patient had an increased caspase-1 activation and IL-1β secretion with or without LPS-stimulation and that CGD patients had uncontrolled inflammation (28, 52). Our observations from the current study and those published results, therefore, suggest that normal NADPH oxidase activity not only plays an important role in killing bacteria but also in downregulating TLR signaling-mediated responses and IL-1β production.

In summary, in the current study, we utilized the approaches that involved infecting mice with *Salmonella* that causes both systemic and mucosal infection, as well as *in vitro* infection of macrophages, to investigate the function of p40*^phox^* in macrophages during bacterial infection. Our data demonstrate that p40*^phox^* is critical for host resistance and survival during *Salmonella* infection and bacteria-mediated intestinal inflammation. One of the major complications of CGD is colitis, which may be treated with corticosteroid. A better understanding of the complex interaction between NADPH oxidase, immune defense, intestinal commensal, and enteric bacterial pathogens will provide important information for the development of new management strategies for disease conditions, such as IBD, cystic fibrosis, and so on.

## Ethics Statement

All animal studies were carried out in accordance with the recommendations in the Guide for the Care and Use of Laboratory Animals of the National Institutes of Health. The protocol was approved by the Sub-committee on Research Animal Care of Massachusetts General Hospital (animal welfare assurance number A3596-01).

## Author Contributions

All the authors reviewed and approved the final version of the manuscript and agreed to be accountable for the content of the work. YL and ML performed most experimental work, acquired and analyzed results, and YL wrote the first draft of the manuscript. CS, SL, and WZ performed experimental work and analyzed results. KC and RX generated and provided gene knockout animals, and analyzed and interpreted the data. WL, RX, and HS designed, supervised, and interpreted the experimental data, critically revised the manuscript. HS wrote the final version of the manuscript.

## Conflict of Interest Statement

The authors declare that the research was conducted in the absence of any commercial or financial relationships that could be construed as a potential conflict of interest.

## References

[1] Rabsch W Andrews HL Kingsley RA Prager R Tschäpe H Adams LG *Salmonella enterica* serotype Typhimurium and its host-adapted variants. Infect Immun (2002) 70:2249–55. 10.1128/Iai.70.5.2249-2255.2002 11953356 PMC127920

[2] Srikanth CV Cherayil BJ. Intestinal innate immunity and the pathogenesis of *Salmonella enteritis*. Immunol Res (2007) 37:61–77. 10.1007/BF02686090 17496347 PMC3199302

[3] Taub N Nairz M Hilber D Hess MW Weiss G Huber LA. The late endosomal adaptor p14 is a macrophage host-defense factor against *Salmonella* infection. J Cell Sci (2012) 125:2698–708. 10.1242/jcs.100073 22427693

[4] Barthel M Hapfelmeier S Quintanilla-Martínez L Kremer M Rohde M Hogardt M Pretreatment of mice with streptomycin provides a *Salmonella enterica serovar* Typhimurium colitis model that allows analysis of both pathogen and host. Infect Immun (2003) 71:2839–58. 10.1128/IAI.71.5.2839-2858.2003 12704158 PMC153285

[5] Srikanth CV Wall DM Maldonado-Contreras A Shi HN Zhou D Demma Z *Salmonella* pathogenesis and processing of secreted effectors by caspase-3. Science (2010) 330:390–3. 10.1126/science.1194598 20947770 PMC4085780

[6] Felmy B Songhet P Slack EMC Müller AJ Kremer M Van Maele L NADPH oxidase deficient mice develop colitis and bacteremia upon infection with normally avirulent, TTSS-1- and TTSS-2-deficient *Salmonella* Typhimurium. PLoS One (2013) 8:e77204. 10.1371/journal.pone.0077204 24143212 PMC3797104

[7] Schäppi MG Jaquet V Belli DC Krause KH. Hyperinflammation in chronic granulomatous disease and anti-inflammatory role of the phagocyte NADPH oxidase. Semin Immunopathol (2008) 30(3):255–71. 10.1007/s00281-008-0119-2 18509648

[8] Szabady RL McCormick BA. Control of neutrophil inflammation at mucosal surfaces by secreted epithelial products. Front Immunol (2013) 4:220. 10.3389/fimmu.2013.00220 23914188 PMC3728559

[9] Harrington L Srikanth CV Antony R Shi HN Cherayil BJ. A role for natural killer cells in intestinal inflammation caused by infection with *Salmonella enterica serovar* Typhimurium. FEMS Immunol Med Microbiol (2007) 51:372–80. 10.1111/j.1574-695X.2007.00313.x 17727655 PMC3205980

[10] Prost LR Sanowar S Miller SI. *Salmonella* sensing of anti-microbial mechanisms to promote survival within macrophages. Immunol Rev (2007) 219:55–65. 10.1111/j.1600-065X.2007.00557.x 17850481

[11] Panday A Sahoo MK Osorio D Batra S. NADPH oxidases: an overview from structure to innate immunity-associated pathologies. Cell Mol Immunol (2015) 12:5–23. 10.1038/cmi.2014.89 25263488 PMC4654378

[12] Deffert C Cachat J Krause K-H. Phagocyte NADPH oxidase, chronic granulomatous disease and mycobacterial infections. Cell Microbiol (2014) 16:1168–78. 10.1111/cmi.12322 24916152

[13] Yi L Liu Q Orandle MS Sadiq-Ali S Koontz SM Choi U P47(Phox) directs murine macrophage cell fate decisions. Am J Pathol (2012) 180:1049–58. 10.1016/j.ajpath.2011.11.019 22222227 PMC3349882

[14] Mastroeni P Vazquez-Torres A Fang FC Xu Y Khan S Hormaeche CE Antimicrobial actions of the NADPH phagocyte oxidase and inducible nitric oxide synthase in experimental salmonellosis. II. Effects on microbial proliferation and host survival in vivo. J Exp Med (2000) 192:237–48. 10.1084/jem.192.2.237 10899910 PMC2193252

[15] Vazquez-Torres A Xu Y Jones-Carson J Holden DW Lucia SM Dinauer MC *Salmonella* pathogenicity island 2-dependent evasion of the phagocyte NADPH oxidase. Science (2000) 287:1655–8. 10.1126/science.287.5458.1655 10698741

[16] Dinauer MC Chronic granulomatous disease and other disorders of phagocyte function. Hematology (2005) 1:89–95. 10.1182/asheducation-2005.1.89 16304364

[17] Raad H Paclet MH Boussetta T Kroviarski Y Morel F Quinn MT Regulation of the phagocyte NADPH oxidase activity: phosphorylation of gp91phox/NOX2 by protein kinase C enhances its diaphorase activity and binding to Rac2, p67phox, and p47phox. FASEB J (2009) 23(4):1011–22. 10.1096/fj.08-114553 19028840 PMC2660639

[18] Matute JD Arias AA Wright NA Wrobel I Waterhouse CC Li XJ A new genetic subgroup of chronic granulomatous disease with autosomal recessive mutations in p40 phox and selective defects in neutrophil NADPH oxidase activity. Blood (2009) 114(15):3309–15. 10.1182/blood-2009-07-231498 19692703 PMC2759653

[19] Conway KL Goel G Sokol H Manocha M Mizoguchi E Terhorst C p40*^phox^* expression regulates neutrophil recruitment and function during the resolution phase of intestinal inflammation. J Immunol (2012) 189:3631–40. 10.4049/jimmunol.1103746 22914050 PMC3780969

[20] Ellson CD Davidson K Ferguson GJ O’Connor R Stephens LR Hawkins PT. Neutrophils from p40^phox-/-^ mice exhibit severe defects in NADPH oxidase regulation and oxidant-dependent bacterial killing. J Exp Med (2006) 203(8):1927–37. 10.1084/jem.20052069 16880254 PMC2118373

[21] Rioux JD Xavier RJ Taylor KD Silverberg MS Goyette P Huett A Genome-wide association study identifies new susceptibility loci for Crohn disease and implicates autophagy in disease pathogenesis. Nat Genet (2007) 39:596–604. 10.1038/ng2032 17435756 PMC2757939

[22] Roberts RL Hollis-Moffatt JE Gearry RB Kennedy MA Barclay ML Merriman TR. Confirmation of association of IRGM and NCF4 with ileal Crohn’s disease in a population-based cohort. Genes Immun (2008) 9(6):561–5. 10.1038/gene.2008.49 18580884

[23] Eglinton TW Roberts R Pearson J Barclay M Merriman TR Frizelle FA Clinical and genetic risk factors for perianal Crohn’s disease in a population-based cohort. Am J Gastroenterol (2012) 107:589–96. 10.1038/ajg.2011.437 22158027

[24] Su C-W Cao Y Zhang M Kaplan J Su L Fu Y Helminth infection impairs autophagy-mediated killing of bacterial enteropathogens by macrophages. J Immunol (2012) 189:1459–66. 10.4049/jimmunol.1200484 22732589 PMC3423331

[25] Filomeni G De Zio D Cecconi F. Oxidative stress and autophagy: the clash between damage and metabolic needs. Cell Death Differ (2015) 22:377–88. 10.1038/cdd.2014.150 25257172 PMC4326572

[26] Harijith A Ebenezer DL Natarajan V. Reactive oxygen species at the crossroads of inflammasome and inflammation. Front Physiol (2014) 5:352. 10.3389/fphys.2014.00352 25324778 PMC4179323

[27] Nakahira K Haspel JA Rathinam VA Lee SJ Dolinay T Lam HC Autophagy proteins regulate innate immune responses by inhibiting the release of mitochondrial DNA mediated by the NALP3 inflammasome. Nat Immunol (2011) 12(3):222–30. 10.1038/ni.1980 21151103 PMC3079381

[28] Meissner F Seger RA Moshous D Fischer A Reichenbach J Zychlinsky A. Inflammasome activation in NADPH oxidase defective mononuclear phagocytes from patients with chronic granulomatous disease. Blood (2010) 116:1570–3. 10.1182/blood-2010-01-264218 20495074 PMC2938844

[29] Su L Su C Qi Y Yang G Zhang M Cherayil BJ Coinfection with an intestinal helminth impairs host innate immunity against *Salmonella enterica serovar* Typhimurium and exacerbates intestinal inflammation in mice. Infect Immun (2014) 82:3855–66. 10.1128/IAI.02023-14 24980971 PMC4187801

[30] Miao EA Leaf IA Treuting PM Mao DP Dors M Sarkar A Caspase-1-induced pyroptosis is an innate immune effector mechanism against intracellular bacteria. Nat Immunol (2010) 11:1136–42. 10.1038/ni.1960 21057511 PMC3058225

[31] Agbor TA Demma Z Mrsny RJ Castillo A Boll EJ Mccormick BA. The oxido-reductase enzyme glutathione peroxidase 4 (GPX4) governs *Salmonella* Typhimurium-induced neutrophil transepithelial migration. Cell Microbiol (2014) 16:1339–53. 10.1111/cmi.12290 24617613 PMC4146641

[32] Akihito H Geem D Denning TL. Macrophage isolation from the mouse small and large intestine. Methods Mol Biol (2016) 1422:171–80. 10.1007/978-1-4939-3603-8_16 27246032 PMC4913542

[33] Weng M Huntley D Huang I-F Foye-Jackson O Wang L Sarkissian A Alternatively activated macrophages in intestinal helminth infection: effects on concurrent bacterial colitis. J Immunol (2007) 179:4721–31. 10.4049/jimmunol.179.7.4721 17878371 PMC3208515

[34] Leto TL Geiszt M. Role of Nox family NADPH oxidases in host defense. Antioxid Redox Signal (2006) 8:1549–61. 10.1089/ars.2006.8.1549 16987010

[35] Sanjuan MA Dillon CP Tait SWG Moshiach S Dorsey F Connell S Toll-like receptor signalling in macrophages links the autophagy pathway to phagocytosis. Nature (2007) 450:1253–7. 10.1038/nature06421 18097414

[36] Schumann RR Belka C Reuter D Lamping N Kirschning CJ Weber JR Lipopolysaccharide activates caspase-1 (interleukin-1-converting enzyme) in cultured monocyticand endothelial cells. Blood (1998) 91(2):577–84. 9427712

[37] Campbell EL Bruyninckx WJ Kelly CJ Glover LE McNamee EN Bowers BE Transmigrating neutrophils shape the mucosal microenvironment through localized oxygen depletion to influence resolution of inflammation. Immunity (2014) 40:66–77. 10.1016/j.immuni.2013.11.020 24412613 PMC3951457

[38] Sanmun D Witasp E Jitkaew S Tyurina YY Kagan VE Ahlin A Involvement of a functional NADPH oxidase in neutrophils and macrophages during programmed cell clearance: implications for chronic granulomatous disease. Am J Physiol Cell Physiol (2009) 297:C621–31. 10.1152/ajpcell.00651.2008 19570889

[39] Cox G Crossley J Xing Z. Macrophage engulfment of apoptotic neutrophils contributes to the resolution of acute pulmonary inflammation in vivo. Am J Respir Cell Mol Biol (1995) 12(2):232–7. 10.1165/ajrcmb.12.2.7865221 7865221

[40] van der Heijden J Bosman ES Reynolds LA Finlay BB. Direct measurement of oxidative and nitrosative stress dynamics in *Salmonella* inside macrophages. Proc Natl Acad Sci U S A (2015) 112(2):560–5. 10.1073/pnas.1414569112 25548165 PMC4299202

[41] Lambeth JD Neish AS. Nox enzymes and new thinking on reactive oxygen: a double-edged sword revisited. Annu Rev Pathol (2014) 9:119–45. 10.1146/annurev-pathol-012513-104651 24050626

[42] Bedard K Krause KH. The NOX family of ROS-generating NADPH oxidases: physiology and pathophysiology. Physiol Rev (2007) 87(1):245–313. 10.1152/physrev.00044.2005 17237347

[43] Rada B Leto TL. Oxidative innate immune defenses by Nox/Duox family NADPH oxidases. Contrib Microbiol (2008) 15:164–87. 10.1159/000136357 18511861 PMC2776633

[44] El Hassani RA Benfares N Caillou B Talbot M Sabourin J-C Belotte V Dual oxidase2 is expressed all along the digestive tract. Am J Physiol Gastrointest Liver Physiol (2005) 288:G933–42. 10.1152/ajpgi.00198.2004 15591162

[45] Grasberger H El-Zaatari M Dang DT Merchant JL. Dual oxidases control release of hydrogen peroxide by the gastric epithelium to prevent helicobacter felis infection and inflammation in mice. Gastroenterology (2013) 145:1045–54. 10.1053/j.gastro.2013.07.011 23860501 PMC3805753

[46] Sommer F Bäckhed F. The gut microbiota engages different signaling pathways to induce Duox2 expression in the ileumand colon epithelium. Mucosal Immunol (2015) 8(2):372–9. 10.1038/mi.2014.74 25160818

[47] Lipinski S Till A Sina C Arlt A Grasberger H Schreiber S DUOX2-derived reactive oxygen species are effectors of NOD2-mediated antibacterial responses. J Cell Sci (2009) 122:3522–30. 10.1242/jcs.050690 19759286

[48] Aviello G Knaus UG ROS in gastrointestinal inflammation: rescue or sabotage? Br J Pharmacol (2017) 174(12):1704–18. 10.1111/bph.13428 26758851 PMC5446568

[49] Hayes P Dhillon S O’Neill K Thoeni C Hui KY Elkadri A Defects in nicotinamide-adenine dinucleotide phosphate oxidase genes NOX1 and DUOX2 in very early onset inflammatory bowel disease. Cell Mol Gastroenterol Hepatol (2015) 1:489–502. 10.1016/j.jcmgh.2015.06.005 26301257 PMC4539615

[50] Pircalabioru G Aviello G Kubica M Zhdanov A Paclet MH Brennan L Defensive mutualism rescues NADPH oxidase inactivation in gut infection. Cell Host Microbe (2016) 19:651–63. 10.1016/j.chom.2016.04.007 27173933

[51] Latz E Xiao TS Stutz A. Activation and regulation of the inflammasomes. Nat Rev Immunol (2013) 13:397–411. 10.1038/nri3452 23702978 PMC3807999

[52] van de Veerdonk FL Smeekens SP Joosten LAB Kullberg BJ Dinarello CA van der Meer JWM Reactive oxygen species-independent activation of the IL-1beta inflammasome in cells from patients with chronic granulomatous disease. Proc Natl Acad Sci U S A (2010) 107:3030–3. 10.1073/pnas.0914795107 20133696 PMC2840365

